# Supplementary material for: Olivetol’s Effects on Metabolic State and Gut Microbiota Functionality in Mouse Models of Alimentary Obesity, Diabetes Mellitus Type 1 and 2, and Hypercholesterolemia
Source: Biomedicines. 2025 Jan 13;13(1):183. doi: 10.3390/biomedicines13010183 (PMC11761620; doi:10.3390/biomedicines13010183)
Supplement: Supplementary file 1 [file biomedicines-13-00183-s001.zip › biomedicines-3389522-supplementary.pdf]

Supplementary Table S1. Alpha diversity indexes estimated for gut microbiota communities of different mouse models. Mean and standard deviation (StDev) values are shown.BL/SD, C57Bl6 mice fed a standard diet; BL/HFD, C57Bl6 mice fed a high-fat diet; BL/SD+C5, C57Bl6 mice fed a standard diet along with olivetol supplementation; BL/HFD+C5, C57Bl6 mice fed a high fat diet along with olivetol supplementation; LDLR/SD, *ldlr*(-/-) mice fed a standard diet; LDLR/SD+C5, *ldlr*(-/-) mice fed a standard diet along with olivetol supplementation; NOD/SD, NOD mice fed a standard diet; NOD/SD+C5, NOD mice fed a standard diet along with olivetol supplementation; DB/SD, *db/db* mice fed a standard diet; DB/SD+C5, *db/db* mice fed a standard diet along with olivetol supplementation.

	BL/SD		BL/SD+C5		BL/HFD		BL/HFD+C5	
	Mean	StDev	Mean	StDev	Mean	StDev	Mean	StDev
PD_whole_tree	49,46	4,74	46,68	4,21	38,40	3,36	37,04	1,64
chao1	1370,59	250,01	1530,40	177,78	1251,01	327,81	1259,40	231,19
observed_otus	798,20	74,43	847,67	69,94	666,40	152,86	689,40	98,77
shannon	7,16	0,30	7,10	0,33	6,08	0,40	6,34	0,26
simpson	0,98	0,00	0,98	0,01	0,95	0,03	0,96	0,01

	DB/SD		DB/SD+C5		NOD/SD		NOD/SD+C5		LDLR/SD		LDLR/SD+C5	
	Mean	StDev	Mean	StDev	Mean	StDev	Mean	StDev	Mean	StDev	Mean	StDev
PD_whole_tree	33,25	2,50	35,87	4,32	34,79	3,92	33,40	3,69	32,12	3,13	27,26	4,58
chao1	992,93	225,97	1307,74	282,94	1357,17	328,97	1069,07	328,35	964,47	189,51	794,89	182,75
observed_otus	591,80	123,42	712,60	131,31	717,30	136,44	589,00	146,49	532,40	84,91	460,50	100,59
shannon	5,55	0,93	6,11	0,83	6,23	0,64	5,97	0,54	5,76	0,40	5,28	0,42
simpson	0,91	0,07	0,94	0,05	0,96	0,02	0,95	0,02	0,95	0,01	0,94	0,01

Supplementary Table S2. Differentially abundant metabolic pathways according to PICRUSt analysis of gut microbiota metagenome sequencing data of db/db mice fed a standard diet with or without olivetol supplementation. Differences in the abundance of metabolic pathways of the gut microbiota were estimated due to multiple Mann–Whitney tests; the Q value reflects a false discovery rate of 5%; the mean rank difference values reflect the direction of changes in the abundance of metabolic pathways (values below zero indicate an increased representation of the pathways, while values above zero indicate a decreased representation of the pathways in the microbiota of mice fed a diet with C5 supplementation).

Pathway name	P value	Mean rank diff.	q value
**superpathway of sulfur oxidation (Acidianus ambivalens)**	0,000011	10	0,001213
**chondroitin sulfate degradation I (bacterial)**	0,000011	10	0,001213
**thiazole biosynthesis II (Bacillus)**	0,000011	10	0,001213
**mannan degradation**	0,000011	10	0,001213
**heme biosynthesis II (anaerobic)**	0,000022	9,8	0,001941
**superpathway of thiamin diphosphate biosynthesis II**	0,000206	9	0,015369

Supplementary Table ST3. Differentially abundant metabolic pathways according to PICRUSt analysis of gut microbiota metagenome sequencing data of NOD mice fed a standard diet with or without olivetol supplementation. Differences in the abundance of metabolic pathways of the gut microbiota were estimated due to multiple Mann–Whitney tests; the Q value reflects a false discovery rate of 5%; the mean rank difference values reflect the direction of changes in the abundance of metabolic pathways (values below zero indicate an increased representation of the pathways, while values above zero indicate a decreased

representation of the pathways in the microbiota of mice fed a diet with C5 supplementation).

Pathway name	P value	Mean rank diff.	q value
**reductive acetyl coenzyme A pathway**	0,000011	10	0,00491

Supplementary Table S4. Differentially abundant metabolic pathways according to PICRUSt analysis of gut microbiota metagenome sequencing data of C57Bl6 mice fed a high fat diet with or without olivetol supplementation. Differences in the abundance of metabolic pathways of the gut microbiota were estimated due to multiple Mann–Whitney tests; the Q value reflects a false discovery rate of 5%; the mean rank difference values reflect the direction of changes in the abundance of metabolic pathways (values below zero indicate an increased representation of the pathways, while values above zero indicate a decreased representation of the pathways in the microbiota of mice fed a diet with C5 supplementation).

Pathway name	P value	Mean rank diff.	q value
**reductive acetyl coenzyme A pathway**	0,000011	-10	0,004808
**superpathway of heme biosynthesis from uroporphyrinogen-III**	0,000022	-9,8	0,004808
**superpathway of glycerol degradation to 1,3-propanediol**	0,00013	9,2	0,011539
**chlorophyllide a biosynthesis II (anaerobic)**	0,00013	9,2	0,011539
**chlorophyllide a biosynthesis III (aerobic, light independent)**	0,00013	9,2	0,011539
**ethylmalonyl-CoA pathway**	0,000476	8,6	0,035258
**chlorophyllide a biosynthesis I (aerobic, light-dependent)**	0,000714	8	0,045332
**ppGpp biosynthesis**	0,00105	-8,2	0,046637
**polymyxin resistance**	0,00105	-8,2	0,046637
**TCA cycle VIII (helicobacter)**	0,00105	-8,2	0,046637

Supplementary Table S5. Differentially abundant metabolic pathways according to PICRUSt analysis of gut microbiota metagenome sequencing data of ldlr(-/-) mice fed a high fat diet with or without olivetol supplementation. Differences in the abundance of metabolic pathways of the gut microbiota were estimated due to multiple Mann–Whitney tests; the Q value reflects a false discovery rate of 5%; the mean rank difference values reflect the direction of changes in the abundance of metabolic pathways (values below zero indicate an increased representation of the pathways, while values above zero indicate a decreased representation of the pathways in the microbiota of mice fed a diet with C5 supplementation).

Pathway name	P value	Mean rank diff.	q value
**chlorophyllide a biosynthesis I (aerobic, light-dependent)**	0,000011	-10	0,000171
**reductive acetyl coenzyme A pathway**	0,000011	10	0,000171
**mixed acid fermentation**	0,000011	10	0,000171
**heme biosynthesis II (anaerobic)**	0,000011	10	0,000171
**O-antigen building blocks biosynthesis (E. coli)**	0,000011	10	0,000171
**TCA cycle VI (obligate autotrophs)**	0,000011	10	0,000171
**S-adenosyl-L-methionine cycle I**	0,000011	10	0,000171
**peptidoglycan biosynthesis IV (Enterococcus faecium)**	0,000011	10	0,000171
**pyrimidine deoxyribonucleotides de novo biosynthesis III**	0,000011	10	0,000171
**adenine and adenosine salvage III**	0,000011	10	0,000171

**starch degradation V**	0,000011	10	0,000171
**UDP-2,3-diacetamido-2,3-dideoxy-α-D-mannuronate biosynthesis**	0,000011	10	0,000171
**pyrimidine deoxyribonucleosides salvage**	0,000011	10	0,000171
**superpathway of L-threonine biosynthesis**	0,000011	10	0,000171
**UDP-N-acetyl-D-glucosamine biosynthesis I**	0,000011	10	0,000171
**homolactic fermentation**	0,000022	9,8	0,000171
**gluconeogenesis I**	0,000022	9,8	0,000171
**superpathway of S-adenosyl-L-methionine biosynthesis**	0,000022	9,8	0,000171
**superpathway of L-lysine, L-threonine and L-methionine biosynthesis I**	0,000022	9,8	0,000171
**peptidoglycan biosynthesis I (meso-diaminopimelate containing)**	0,000022	9,8	0,000171
**superpathway of L-isoleucine biosynthesis I**	0,000022	9,8	0,000171
**superpathway of L-methionine biosynthesis (transsulfuration)**	0,000022	9,8	0,000171
**glycolysis II (from fructose 6-phosphate)**	0,000022	9,8	0,000171
**urate biosynthesis/inosine 5'-phosphate degradation**	0,000022	9,8	0,000171
**superpathway of adenosine nucleotides de novo biosynthesis II**	0,000022	9,8	0,000171
**sucrose degradation III (sucrose invertase)**	0,000022	9,8	0,000171
**peptidoglycan biosynthesis III (mycobacteria)**	0,000022	9,8	0,000171
**UDP-N-acetylmuramoyl-pentapeptide biosynthesis II (lysine-containing)**	0,000022	9,8	0,000171
**UDP-N-acetylmuramoyl-pentapeptide biosynthesis I (meso-diaminopimelate containing)**	0,000022	9,8	0,000171
**pyrimidine deoxyribonucleotides de novo biosynthesis II**	0,000022	9,8	0,000171
**superpathway of pyrimidine nucleobases salvage**	0,000022	9,8	0,000171
**superpathway of adenosine nucleotides de novo biosynthesis I**	0,000022	9,8	0,000171
**NAD salvage pathway I**	0,000022	9,8	0,000171
**tRNA charging**	0,000022	9,8	0,000171
**glycolysis III (from glucose)**	0,000043	9,6	0,000179
**L-arginine biosynthesis I (via L-ornithine)**	0,000043	9,6	0,000179
**L-arginine biosynthesis II (acetyl cycle)**	0,000043	9,6	0,000179
**colanic acid building blocks biosynthesis**	0,000043	9,6	0,000179
**dTDP-L-rhamnose biosynthesis I**	0,000043	9,6	0,000179
**fatty acid elongation -- saturated**	0,000043	9,6	0,000179
**glycolysis I (from glucose 6-phosphate)**	0,000043	9,6	0,000179
**L-methionine biosynthesis I**	0,000043	9,6	0,000179
**methylerythritol phosphate pathway I**	0,000043	9,6	0,000179
**Bifidobacterium shunt**	0,000043	9,6	0,000179
**polyisoprenoid biosynthesis (E. coli)**	0,000043	9,6	0,000179
**L-lysine biosynthesis III**	0,000043	9,6	0,000179
**pyruvate fermentation to acetate and lactate II**	0,000043	9,6	0,000179
**superpathway of geranylgeranyl diphosphate biosynthesis II (via MEP)**	0,000043	9,6	0,000179
**superpathway of bacteriochlorophyll a biosynthesis**	0,000043	-9,6	0,000179
**GDP-mannose biosynthesis**	0,000043	9,6	0,000179
**UMP biosynthesis**	0,000043	9,6	0,000179
**5-aminoimidazole ribonucleotide biosynthesis I**	0,000043	9,6	0,000179
**5-aminoimidazole ribonucleotide biosynthesis II**	0,000043	9,6	0,000179

**inosine-5'-phosphate biosynthesis I**	0,000043	9,6	0,000179
**superpathway of 5-aminoimidazole ribonucleotide biosynthesis**	0,000043	9,6	0,000179
**galactose degradation I (Leloir pathway)**	0,000043	9,6	0,000179
**superpathway of glucose and xylose degradation**	0,000043	9,6	0,000179
**adenosine ribonucleotides de novo biosynthesis**	0,000043	9,6	0,000179
**adenosine deoxyribonucleotides de novo biosynthesis II**	0,000043	9,6	0,000179
**guanosine ribonucleotides de novo biosynthesis**	0,000043	9,6	0,000179
**guanosine deoxyribonucleotides de novo biosynthesis II**	0,000043	9,6	0,000179
**L-arginine biosynthesis IV (archaebacteria)**	0,000043	9,6	0,000179
**methylerythritol phosphate pathway II**	0,000043	9,6	0,000179
**anhydromuropeptides recycling**	0,000043	9,6	0,000179
**aspartate superpathway**	0,000043	9,6	0,000179
**chorismate biosynthesis I**	0,000076	9,4	0,000261
**superpathway of aromatic amino acid biosynthesis**	0,000076	9,4	0,000261
**L-methionine biosynthesis III**	0,000076	9,4	0,000261
**heterolactic fermentation**	0,000076	9,4	0,000261
**superpathway of phospholipid biosynthesis I (bacteria)**	0,000076	9,4	0,000261
**L-isoleucine biosynthesis III**	0,000076	9,4	0,000261
**chorismate biosynthesis from 3-dehydroquinate**	0,000076	9,4	0,000261
**TCA cycle V (2-oxoglutarate:ferredoxin oxidoreductase)**	0,000076	9,4	0,000261
**pyruvate fermentation to isobutanol (engineered)**	0,000076	9,4	0,000261
**superpathway of L-alanine biosynthesis**	0,000076	9,4	0,000261
**phosphatidylglycerol biosynthesis I (plastidic)**	0,000076	9,4	0,000261
**phosphatidylglycerol biosynthesis II (non-plastidic)**	0,000076	9,4	0,000261
**NAD biosynthesis I (from aspartate)**	0,000076	9,4	0,000261
**superpathway of branched amino acid biosynthesis**	0,00013	9,2	0,000368
**Calvin-Benson-Bassham cycle**	0,00013	9,2	0,000368
**L-lysine biosynthesis I**	0,00013	9,2	0,000368
**L-ornithine biosynthesis**	0,00013	9,2	0,000368
**glycogen degradation I (bacterial)**	0,00013	9,2	0,000368
**L-histidine biosynthesis**	0,00013	9,2	0,000368
**L-isoleucine biosynthesis I (from threonine)**	0,00013	9,2	0,000368
**L-leucine degradation I**	0,00013	9,2	0,000368
**pentose phosphate pathway (non-oxidative branch)**	0,00013	9,2	0,000368
**acetylene degradation**	0,00013	9,2	0,000368
**pentose phosphate pathway**	0,00013	9,2	0,000368
**L-isoleucine biosynthesis II**	0,00013	9,2	0,000368
**CDP-diacylglycerol biosynthesis I**	0,00013	9,2	0,000368
**preQ0 biosynthesis**	0,00013	9,2	0,000368
**CDP-diacylglycerol biosynthesis II**	0,00013	9,2	0,000368
**peptidoglycan maturation (meso-diaminopimelate containing)**	0,00013	9,2	0,000368
**L-valine biosynthesis**	0,00013	9,2	0,000368
**coenzyme A biosynthesis I**	0,000206	9	0,000558
**superpathway of sulfur oxidation (Acidianus ambivalens)**	0,000206	-9	0,000558

6-hydroxymethyl-dihydropterin diphosphate biosynthesis I	0,000206	9	0,000558
TCA cycle I (prokaryotic)	0,000206	9	0,000558
superpathway of tetrahydrofolate biosynthesis and salvage	0,000325	8,8	0,000808
glycogen biosynthesis I (from ADP-D-Glucose)	0,000325	8,8	0,000808
L-lysine fermentation to acetate and butanoate	0,000325	8,8	0,000808
incomplete reductive TCA cycle	0,000325	8,8	0,000808
L-lysine biosynthesis VI	0,000325	8,8	0,000808
L-isoleucine biosynthesis IV	0,000325	8,8	0,000808
allantoin degradation to glyoxylate III	0,000325	8,8	0,000808
superpathway of tetrahydrofolate biosynthesis	0,000325	8,8	0,000808
superpathway of GDP-mannose-derived O-antigen building blocks biosynthesis	0,000325	8,8	0,000808
N10-formyl-tetrahydrofolate biosynthesis	0,000487	8,6	0,001149
methanogenesis from acetate	0,000487	8,6	0,001149
L-lysine biosynthesis II	0,000487	8,6	0,001149
L-arginine biosynthesis III (via N-acetyl-L-citrulline)	0,000487	8,6	0,001149
cis-vaccenate biosynthesis	0,000487	8,6	0,001149
gondoate biosynthesis (anaerobic)	0,000487	8,6	0,001149
superpathway of purine nucleotides de novo biosynthesis II	0,000725	8,4	0,001572
superpathway of menaquinol-9 biosynthesis	0,000725	8,4	0,001572
superpathway of menaquinol-6 biosynthesis I	0,000725	8,4	0,001572
superpathway of demethylmenaquinol-6 biosynthesis I	0,000725	8,4	0,001572
superpathway of demethylmenaquinol-9 biosynthesis	0,000725	8,4	0,001572
superpathway of menaquinol-10 biosynthesis	0,000725	8,4	0,001572
thiamin salvage II	0,000725	8,4	0,001572
inosine-5'-phosphate biosynthesis III	0,000725	8,4	0,001572
superpathway of L-serine and glycine biosynthesis I	0,000725	8,4	0,001572
superpathway of thiamin diphosphate biosynthesis I	0,000725	8,4	0,001572
pantothenate and coenzyme A biosynthesis I	0,00105	8,2	0,002205
L-1,2-propanediol degradation	0,00105	8,2	0,002205
superpathway of pyrimidine deoxyribonucleoside salvage	0,00105	8,2	0,002205
dTDP-N-acetylthomosamine biosynthesis	0,00105	8,2	0,002205
GDP-D-glycero-α-D-manno-heptose biosynthesis	0,001505	8	0,003018
queuosine biosynthesis	0,001505	8	0,003018
superpathway of UDP-N-acetylglucosamine-derived O-antigen building blocks biosynthesis	0,001505	8	0,003018
superpathway of purine deoxyribonucleosides degradation	0,001505	8	0,003018
superpathway of pyrimidine deoxyribonucleosides degradation	0,001505	8	0,003018
TCA cycle VIII (helicobacter)	0,001505	8	0,003018
superpathway of L-aspartate and L-asparagine biosynthesis	0,002089	7,8	0,003873
pyruvate fermentation to butanoate	0,002089	7,8	0,003873
phosphopantothenate biosynthesis I	0,002089	7,8	0,003873
4-aminobutanoate degradation V	0,002089	7,8	0,003873
sucrose degradation IV (sucrose phosphorylase)	0,002089	7,8	0,003873
acetyl-CoA fermentation to butanoate II	0,002089	7,8	0,003873

**superpathway of geranylgeranyldiphosphate biosynthesis I (via mevalonate)**	0,002089	7,8	0,003873
**superpathway of Clostridium acetobutylicum acidogenic fermentation**	0,002089	7,8	0,003873
**thiazole biosynthesis I (E. coli)**	0,002089	7,8	0,003873
**mevalonate pathway I**	0,002089	7,8	0,003873
**L-tryptophan biosynthesis**	0,002089	7,8	0,003873
**fatty acid β-oxidation I**	0,002879	7,6	0,005265
**glutaryl-CoA degradation**	0,002879	7,6	0,005265
**factor 420 biosynthesis**	0,003096	-7	0,005623
**hexitol fermentation to lactate, formate, ethanol and acetate**	0,003886	7,4	0,006918
**CMP-legionaminate biosynthesis I**	0,003886	7,4	0,006918
**superpathway of pyrimidine deoxyribonucleotides de novo biosynthesis (E. coli)**	0,003886	7,4	0,006918
**chlorophyllide a biosynthesis II (anaerobic)**	0,004871	-7,2	0,008558
**chlorophyllide a biosynthesis III (aerobic, light independent)**	0,004871	-7,2	0,008558
**tetrapyrrole biosynthesis I (from glutamate)**	0,005196	7,2	0,008953
**6-hydroxymethyl-dihydropterin diphosphate biosynthesis III (Chlamydia)**	0,005196	7,2	0,008953
**flavin biosynthesis I (bacteria and plants)**	0,005196	7,2	0,008953
**L-histidine degradation I**	0,006841	7	0,011494
**pyruvate fermentation to propanoate I**	0,006841	7	0,011494
**aerobic respiration I (cytochrome c)**	0,006841	7	0,011494
**superpathway of purine nucleotides de novo biosynthesis I**	0,006841	7	0,011494
**tRNA processing**	0,008931	-6,8	0,01491
**methylaspartate cycle**	0,011258	6,4	0,01868
**purine nucleotides degradation II (aerobic)**	0,011496	6,6	0,018728
**glycerol degradation to butanol**	0,011496	6,6	0,018728
**purine ribonucleosides degradation**	0,011496	6,6	0,018728
**coenzyme M biosynthesis I**	0,012622	6	0,020439
**nitrate reduction I (denitrification)**	0,014083	6	0,022668
**superpathway of arginine and polyamine biosynthesis**	0,01469	6,4	0,022693
**D-galacturonate degradation I**	0,01469	6,4	0,022693
**superpathway of polyamine biosynthesis II**	0,01469	6,4	0,022693
**superpathway of polyamine biosynthesis I**	0,01469	6,4	0,022693
**superpathway of L-phenylalanine biosynthesis**	0,01469	-6,4	0,022693
**superpathway of L-tyrosine biosynthesis**	0,01469	-6,4	0,022693
**adenosine nucleotides degradation II**	0,01469	6,4	0,022693
**superpathway of N-acetylglucosamine, N-acetylmannosamine and N-acetylneuraminate degradation**	0,018543	6,2	0,028161
**toluene degradation I (aerobic) (via o-cresol)**	0,018543	6,2	0,028161
**toluene degradation II (aerobic) (via 4-methylcatechol)**	0,018543	6,2	0,028161
**lipid IVA biosynthesis**	0,023231	6	0,034499
**CMP-3-deoxy-D-manno-octulosonate biosynthesis I**	0,023231	6	0,034499
**Kdo transfer to lipid IVA III (Chlamydia)**	0,023231	6	0,034499
**myo-, chiro- and scillo-inositol degradation**	0,023231	-6	0,034499
**4-hydroxyphenylacetate degradation**	0,027723	5,4	0,040945

**arginine, ornithine and proline interconversion**	0,028806	5,8	0,041629
**superpathway of hexuronide and hexuronate degradation**	0,028806	5,8	0,041629
**superpathway of β-D-glucuronide and D-glucuronate degradation**	0,028806	5,8	0,041629
**catechol degradation I (meta-cleavage pathway)**	0,028806	5,8	0,041629
**superpathway of phenylethylamine degradation**	0,032508	5	0,046728
**catechol degradation III (ortho-cleavage pathway)**	0,033958	5,4	0,047928
**aromatic compounds degradation via β-ketoadipate**	0,033958	5,4	0,047928
**superpathway of salicylate degradation**	0,033958	5,4	0,047928
**toluene degradation IV (aerobic) (via catechol)**	0,034056	5	0,047928
**TCA cycle IV (2-oxoglutarate decarboxylase)**	0,035463	5,6	0,048144
**L-glutamate degradation V (via hydroxyglutarate)**	0,035463	5,6	0,048144
**palmitate biosynthesis II (bacteria and plants)**	0,035463	5,6	0,048144
**chondroitin sulfate degradation I (bacterial)**	0,035463	-5,6	0,048144
**pyruvate fermentation to acetone**	0,035463	5,6	0,048144
**aromatic biogenic amine degradation (bacteria)**	0,035463	5,6	0,048144
**superpathway of pyrimidine ribonucleotides de novo biosynthesis**	0,035463	5,6	0,048144

Supplementary Table S6. Differentially abundant metabolic pathways according to PICRUSt analysis of gut microbiota metagenome sequencing data of C57Bl6 mice fed a standard diet with or without olivetol supplementation. Differences in the abundance of metabolic pathways of the gut microbiota were estimated due to multiple Mann–Whitney tests; the Q value reflects a false discovery rate of 5%; the mean rank difference values reflect the direction of changes in the abundance of metabolic pathways (values below zero indicate an increased representation of the pathways, while values above zero indicate a decreased representation of the pathways in the microbiota of mice fed a diet with C5 supplementation).

Pathway name	P value	Mean rank diff.	q value
**adenosylcobalamin biosynthesis from cobyrinate a,c-diamide I**	0,000043	9,289	0,008388
**adenosylcobalamin salvage from cobinamide II**	0,000043	9,289	0,008388
**adenosylcobalamin salvage from cobinamide I**	0,000087	9,078	0,008388
**L-glutamate and L-glutamine biosynthesis**	0,000087	9,078	0,008388
**L-lysine biosynthesis I**	0,00026	8,656	0,010625
**glucose and glucose-1-phosphate degradation**	0,00026	8,656	0,010625
**tetrapyrrole biosynthesis II (from glycine)**	0,00026	8,656	0,010625
**pyruvate fermentation to isobutanol (engineered)**	0,00026	8,656	0,010625
**NAD salvage pathway I**	0,00026	8,656	0,010625
**L-arginine biosynthesis II (acetyl cycle)**	0,000411	8,444	0,010625
**L-ornithine biosynthesis**	0,000411	8,444	0,010625
**glycogen degradation I (bacterial)**	0,000411	8,444	0,010625
**starch degradation V**	0,000411	8,444	0,010625
**phosphatidylglycerol biosynthesis I (plastidic)**	0,000411	8,444	0,010625
**phosphatidylglycerol biosynthesis II (non-plastidic)**	0,000411	8,444	0,010625
**methanogenesis from acetate**	0,00065	8,233	0,014411
**D-fructuronate degradation**	0,00065	8,233	0,014411

**chorismate biosynthesis I**	0,000974	8,022	0,014411
**Calvin-Benson-Bassham cycle**	0,000974	8,022	0,014411
**superpathway of aromatic amino acid biosynthesis**	0,000974	8,022	0,014411
**fucose degradation**	0,000974	8,022	0,014411
**L-histidine degradation I**	0,000974	-8,022	0,014411
**purine nucleobases degradation I (anaerobic)**	0,000974	8,022	0,014411
**S-adenosyl-L-methionine cycle I**	0,000974	8,022	0,014411
**peptidoglycan maturation (meso-diaminopimelate containing)**	0,000974	8,022	0,014411
**L-arginine biosynthesis I (via L-ornithine)**	0,001451	7,811	0,014411
**glycogen biosynthesis I (from ADP-D-Glucose)**	0,001451	7,811	0,014411
**L-isoleucine biosynthesis I (from threonine)**	0,001451	7,811	0,014411
**pentose phosphate pathway (non-oxidative branch)**	0,001451	7,811	0,014411
**superpathway of phospholipid biosynthesis I (bacteria)**	0,001451	7,811	0,014411
**L-isoleucine biosynthesis II**	0,001451	7,811	0,014411
**L-isoleucine biosynthesis III**	0,001451	7,811	0,014411
**L-isoleucine biosynthesis IV**	0,001451	7,811	0,014411
**sucrose degradation III (sucrose invertase)**	0,001451	7,811	0,014411
**galactose degradation I (Leloir pathway)**	0,001451	7,811	0,014411
**L-arginine biosynthesis IV (archaebacteria)**	0,001451	7,811	0,014411
**purine ribonucleosides degradation**	0,001451	7,811	0,014411
**superpathway of pyrimidine deoxyribonucleosides degradation**	0,001451	7,811	0,014411
**L-valine biosynthesis**	0,001451	7,811	0,014411
**L-lysine biosynthesis VI**	0,0021	7,6	0,018923
**chorismate biosynthesis from 3-dehydroquinate**	0,0021	7,6	0,018923
**superpathway of purine deoxyribonucleosides degradation**	0,0021	7,6	0,018923
**L-tryptophan biosynthesis**	0,0021	7,6	0,018923
**superpathway of branched amino acid biosynthesis**	0,002988	7,389	0,022698
**superpathway of hexuronide and hexuronate degradation**	0,002988	7,389	0,022698
**superpathway of N-acetylglucosamine, N-acetylmannosamine and N-acetylneuraminate degradation**	0,002988	7,389	0,022698
**superpathway of β-D-glucuronide and D-glucuronate degradation**	0,002988	7,389	0,022698
**CDP-diacylglycerol biosynthesis I**	0,002988	7,389	0,022698
**dTDP-N-acetylthomosamine biosynthesis**	0,002988	7,389	0,022698
**CDP-diacylglycerol biosynthesis II**	0,002988	7,389	0,022698
**superpathway of L-threonine biosynthesis**	0,002988	7,389	0,022698
**L-tryptophan degradation XII (Geobacillus)**	0,003432	7,178	0,025568
**glycolysis III (from glucose)**	0,004135	7,178	0,027624
**superpathway of L-aspartate and L-asparagine biosynthesis**	0,004135	7,178	0,027624
**acetylene degradation**	0,004135	7,178	0,027624
**glycolysis V (Pyrococcus)**	0,004135	7,178	0,027624
**L-lysine biosynthesis III**	0,004135	7,178	0,027624
**thiamin salvage II**	0,004135	7,178	0,027624
**N10-formyl-tetrahydrofolate biosynthesis**	0,005672	6,967	0,03434
**methylerythritol phosphate pathway I**	0,005672	6,967	0,03434

**superpathway of L-isoleucine biosynthesis I**	0,005672	6,967	0,03434
**tetrapyrrole biosynthesis I (from glutamate)**	0,005672	6,967	0,03434
**UMP biosynthesis**	0,005672	6,967	0,03434
**methylerythritol phosphate pathway II**	0,005672	6,967	0,03434
**O-antigen building blocks biosynthesis (E. coli)**	0,007621	6,756	0,042793
**GDP-D-glycero-α-D-manno-heptose biosynthesis**	0,007621	6,756	0,042793
**adenosine ribonucleotides de novo biosynthesis**	0,007621	6,756	0,042793
**gondoate biosynthesis (anaerobic)**	0,007621	6,756	0,042793
**tRNA charging**	0,007621	6,756	0,042793

Supplementary Table S7. Primers used to amplify the V3-V4 region of bacterial 16S rRNA gene.

Forward Primer:	**TCGTCGGCAGCGTCAGATGTGTATAAGAGACAG**CCTACGGGAGGCAGCAG
Reverse Primer:	**GTCTCGTGGGCTCGGAGATGTGTATAAGAGACAG**GACTACAAGGGTATCTAATCC
